# Semi-Automated and Direct Localization and Labeling of EEG Electrodes Using MR Structural Images for Simultaneous fMRI-EEG

**DOI:** 10.3389/fnins.2020.558981

**Published:** 2020-12-22

**Authors:** Abhishek S. Bhutada, Pradyumna Sepúlveda, Rafael Torres, Tomás Ossandón, Sergio Ruiz, Ranganatha Sitaram

**Affiliations:** ^1^Department of Molecular and Cellular Biology, University of California, Berkeley, Berkeley, CA, United States; ^2^Institute of Cognitive Neuroscience, University College London, London, United Kingdom; ^3^Department of Psychiatry, Faculty of Medicine, Interdisciplinary Center for Neuroscience, Pontificia Universidad Católica de Chile, Santiago, Chile; ^4^Laboratory for Brain–Machine Interfaces and Neuromodulation, Pontificia Universidad Católica de Chile, Santiago, Chile; ^5^Institute for Biological and Medical Engineering, Pontificia Universidad Católica de Chile, Santiago, Chile

**Keywords:** electroencephalography, magnetic resonance imaging, EEG/fMRI, source localization, electrode positioning, electrode labeling

## Abstract

Electroencephalography (EEG) source reconstruction estimates spatial information from the brain’s electrical activity acquired using EEG. This method requires accurate identification of the EEG electrodes in a three-dimensional (3D) space and involves spatial localization and labeling of EEG electrodes. Here, we propose a new approach to tackle this two-step problem based on the simultaneous acquisition of EEG and magnetic resonance imaging (MRI). For the step of spatial localization of electrodes, we extract the electrode coordinates from the curvature of the protrusions formed in the high-resolution T1-weighted brain scans. In the next step, we assign labels to each electrode based on the distinguishing feature of the electrode’s distance profile in relation to other electrodes. We then compare the subject’s electrode data with template-based models of prelabeled distance profiles of correctly labeled subjects. Based on this approach, we could localize EEG electrodes in 26 head models with over 90% accuracy in the 3D localization of electrodes. Next, we performed electrode labeling of the subjects’ data with progressive improvements in accuracy: with ∼58% accuracy based on a single EEG-template, with ∼71% accuracy based on 3 EEG-templates, and with ∼76% accuracy using 5 EEG-templates. The proposed semi-automated method provides a simple alternative for the rapid localization and labeling of electrodes without the requirement of any additional equipment than what is already used in an EEG-fMRI setup.

## Introduction

Despite the usefulness of electroencephalography (EEG) to study the dynamic changes in brain signal, one of its historical weaknesses has been its restricted spatial resolution. An approach to tackle this issue has been to analyze EEG signals with an inverse mathematical model and trace brain activity through a method called EEG source reconstruction (Michel et al., 2004). By using models of the brain’s structure, the inverse model allows us to locate regions that are activated over time from the information given by the voltage measurements of the electrodes. EEG source reconstruction serves many clinical and neuroscientific purposes such as epileptic seizure mapping and understanding neurovascular coupling (Gavaret et al., 2004; Vulliemoz et al., 2010; Yuan et al., 2010; Hanslmayr et al., 2011; De Ciantis and Lemieux, 2013; Lei et al., 2015). On the other hand, another way to deal with EEG drawbacks has been relying on multimodal approaches. The development of EEG-fMRI has resulted in an interesting symbiosis of two techniques that allow a richer and more comprehensive understanding of brain dynamics in a non-invasive way (Goebel and Esposito, 2009; Huster et al., 2012).

For a successful source reconstruction analysis of EEG signals, it is necessary to precisely obtain three-dimensional (3D) coordinates of the location of each electrode for each individual subject (Dalal et al., 2014). Although some previous approaches have relied on a standard positioning of electrodes in the EEG cap, the personalization of electrode mapping for each experimental subject improves the accuracy of the observed results by taking into consideration the differences in head shape and size across subjects. Although some external devices and methods have been proposed to obtain electrode locations (see below), in the present work we propose one method to use the standard EEG-fMRI experimental setup, i.e., MR-compatible EEG system with and electrode cap using conductive gel plus a magnetic resonance imaging (MRI) scanner, without further additions or extra MR-sequences to obtain EEG positions and labeling, facilitating a more accurate analysis of EEG in a multimodal environment.

The process of spatial localization of EEG electrodes involves two major steps: (1) correctly localizing and obtaining the 3D coordinates of each electrode, and (2) distinguishing each electrode by finding its proper label. There have been attempts to solve these two steps of the problem (De Munck et al., 1991; Steddin and Botzel, 1995; Yoo et al., 1997; Le et al., 1998; Koessler et al., 2007; Péchaud et al., 2007); however, there are only a few approaches that have automated this process of mapping electrodes. In the following paragraphs, we summarize the existing manual, semi-automated, and automated methods of EEG localization and labeling.

### Manual Localization and Labeling

The most rudimentary methods are based on direct manual measurements (e.g., calipers or compass) of the distances between each electrode and particular landmarks to later calculate the Cartesian coordinates using a system of equations (De Munck et al., 1991). Recent methods through the use of digitizers, cameras, or external devices allow for manual localization of electrodes on the standard cap (Koessler et al., 2007). Electromagnetic digitization utilizes an electromagnetic field transmitter and multiple receivers across the subject’s head in order to create a model. Another stylus receiver is then used as a way to manually localize electrodes on the head (Le et al., 1998). An ultrasound digitizer uses a similar method for digitizing the subject’s head and for localizing EEG electrodes by using sound impulses (Steddin and Botzel, 1995). A photogrammetry system, also known as geodesic photogrammetry system (GPS), uses a system of multiple cameras placed in a polyhedron-based structure around the subject’s head and allows for 3D reconstruction of a head model and localization of the electrodes through method of triangulation (Russel et al., 2005; Clausner et al., 2017). The photogrammetry method still involves manual selection of points on each of the pictures taken. While these methods are useful in visually generating a head model, the process of localizing and labeling each of the electrodes is still manual and time-consuming. Some methods have taken advantage of the manual digitization and they have co-registered it with MRI volumes, using fiducial points and surface matching (Brinkmann et al., 1998; Lamm et al., 2001).

### Semi-Automated Localization and Labeling

**FIGURE 1:**
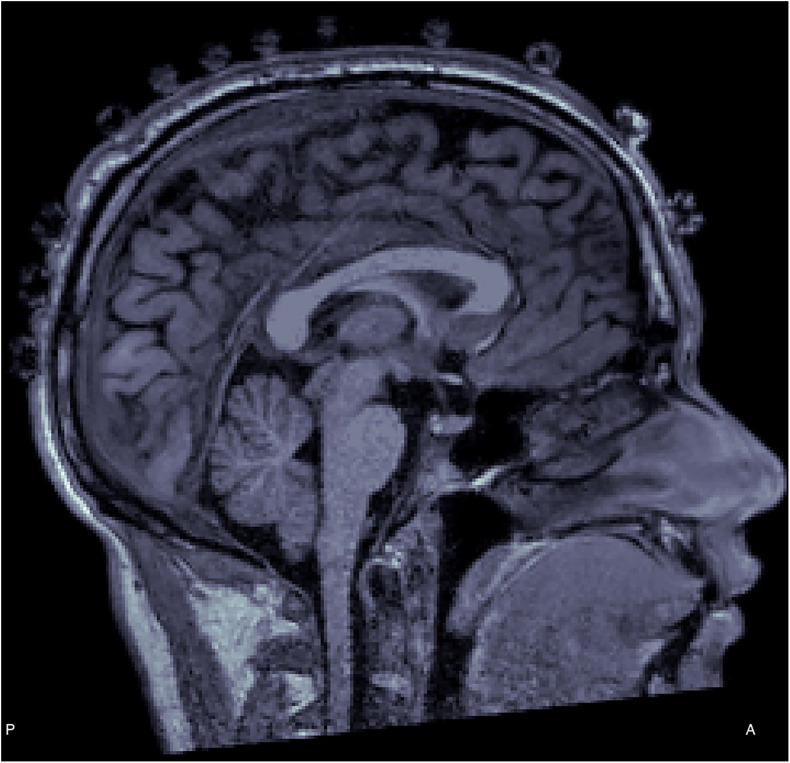
T1-weighted (T1W) structural image with electrode protrusions appearing over the scalp.

Initial attempts to make visible electrodes in MR have considered the inclusion of additional tags e.g., inclusion of gadolinium capsules, and manual segmentation of the electrodes from the images (Yoo et al., 1997). However, other methods have relied on the fact that electrodes are visible thanks to the conductive gel in some structural brain images acquired using MRI (see Figure 1). Using this feature, a semi-automated method of localizing electrodes, the Pancake View Method, has been implemented (De Munck et al., 2011). In this method, a flat pancake view of the head can be derived from T1-weighted (T1W) structural images. Each electrode artifact can be visualized in a single two-dimensional (2D) view. Later, locations and labels have to be selected manually for each electrode, generating a grid with known vertices. Using the template grid, the electrodes can be labeled automatically on other subjects using the same cap. Finally, the 2D coordinates of the pancake view are transformed to obtain the 3D coordinates of each one of the electrodes in the MRI coordinate frame. Another method based on MR, allows the localization of electrodes without relying on the presence of conductive gel, but using additional MR-sequences ultra-short echo time sequences (UTE) exclusively sensitive to the polymer material of the electrodes (Butler et al., 2017).

The combinatorial optimization and self-calibration is a different semi-automated approach to localizing electrode positions (Péchaud et al., 2007). In this method, the 3D coordinates of each electrode are reconstructed with a photogrammetry-based method of ten different pictures of the subject’s head from various angles. The 3D coordinates are used to generate a template head with labels. To label the electrodes on a test subject, a minimization algorithm is then applied on the coordinates of the subject’s head and the previously prepared template head in order to automatically provide labels for each of the electrodes.

These methods are closer to fully automatic approaches to solving the two-step problem; however, a limitation to these techniques is that they both require manual selection of points to localize the electrodes which can be quite time-consuming.

### Fully-Automated Localization and Labeling

A method for automated localization and labeling of electrodes also exploits the protrusions pertaining to EEG electrodes that appear in MR structural images (Koessler et al., 2008). In this method, structural MR images are pre-processed to enhance the clarity of the electrode protrusions so that they are more clearly visible as high-intensity voxels. With this approach, electrodes can be localized more effectively by segmenting the different layers of the head and identifying the voxels with the highest intensity across the scalp of the subject. After the electrodes are localized, a point drift method is used to register and label each electrode (Koessler et al., 2008). Although this approach results in automated localization and labeling, it requires specific sensors for detection of EEG electrodes in MR anatomical images. These sensors are not commonly used on a standard 64 channel EEG cap and must be externally glued on the subject’s scalp.

Marino et al. (2016) reported an automatic method devised for high-density electrode caps, which extracts the electrode position through image processing and labels the electrodes using a transformation of the candidate position to MNI space to be matched with a template of the desired EEG positions. Another method (Fleury et al., 2019) allows the localization of electrodes without relying on the presence of conductive gel (although it uses additional UTE sequences, mentioned above) and implements automatic labeling using the iterative Closest Point algorithm, over a template of the electrode cap. These approaches again rely on the use of specialized electrodes for localization.

In another recent study, the same high-density electrode caps used in Marino’s study were localized with the use of 3D scanners (Taberna et al., 2019). The approach used in Taberna’s study was accurate in localization and used Closest Point algorithm to label the electrodes. Another study shows how 3D scanners have improved EEG source modeling due to a more reliable electrode localization (Homölle and Oostenveld, 2019). Yet, these approaches require the additional hardware (i.e., 3D scanner) in order to localize electrodes.

### The Proposed Approach

**FIGURE 2:**
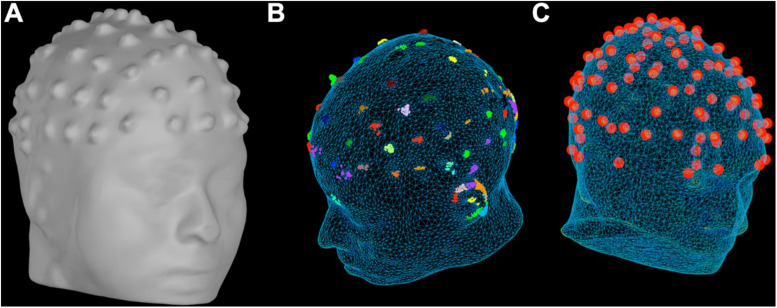
Head Models. **(A)** Head model generated from the T1W structural scan of a subject using Free Surfer. Each protrusion on the scalp is an EEG electrode. **(B)** Clusters of vertices with high curvature are used to generate the position of the potential electrodes. Different clusters are depicted using different colors. **(C)** Using the curvature information of the head model, we can identify electrodes using the proposed method.

Our MR-based method provides a direct way of solving this two-step problem of localization and labeling of the electrodes in a simultaneous EEG/MRI setup. With our approach, the user does not have the need for additional equipment (e.g., digitizers and cameras) or the need for specialized MR sequences (e.g., UTE sequences) to solve this two-step problem (Steddin and Botzel, 1995; Yoo et al., 1997; Le et al., 1998; Russel et al., 2005; Koessler et al., 2007; Péchaud et al., 2007; Marino et al., 2016; Butler et al., 2017; Clausner et al., 2017; Fleury et al., 2019; Homölle and Oostenveld, 2019; Taberna et al., 2019). We take advantage of the fact that standard EEG electrodes can be seen in the standard high-resolution MRI structural images. A surface model of the head is first generated from the T1 images and electrodes are localized in the Cartesian coordinate system by considering the fact that each electrode protrusion in the MRI images possesses relatively higher curvature than the surrounding scalp in the generated head mesh (see Figure 2). In order to isolate the specific 3D coordinate value of a particular electrode, we consider the centroid of groups of points with maximal curvature to obtain the electrode location. The locations of all electrodes are determined in this manner first. In the next step, electrodes are labeled based on the idea that each electrode has a particular set of distances to all of the other electrodes. The set of all distances from each electrode to all other electrodes is called the Distance Profile (see Figure 4). The approach is based on the assumption that for a given EEG cap configuration, distance profiles should be constant despite changes in the point of reference of the coordinates system or in the shape of experimental subject heads. In this way we present an approach that provides a simple solution to the problem of localizing and labeling electrodes.

## Materials and Methods

Our method involves pre-processing of the T1W structural images in order to create a head model that can be used to extract the electrode positions. Next, the localization of electrodes is performed by finding vertices in the head mesh which have maximal curvature. The final step involves utilizing the distance profile criterion in order to assign labels to each of the electrode positions. An approximate duration of applying our method to localize and label electrodes is around 10 min per electrode set (participant), mostly giving time required for human intervention (i.e., indicating fiducial points in MR, pruning any extraneous electrodes located, etc.). Our study considers 26 T1W structural scans (subjects’ ages: 22.86 ± 1.54) acquired during real-time fMRI neurofeedback study during simultaneous acquisition of EEG signals fMRI results from that study can be found in Sepulveda et al. (2016). The experimental protocol was approved by the ethics committee of Pontificia Universidad Católica de Chile. Each participant signed a written informed consent during the study. More details on the approach are available in the Supplementary Methods.

### MR and EEG Acquisition

MR acquisition was done using a Philips Achieva 1.5T MR scanner (Philips Healthcare, Best, Netherlands) at the Pontificia Universidad Católica de Chile. A standard 8-channel head coil was used. Structural T1W brain volumes were acquired using T1W-3D Turbo Field Echo (TFE, magnetization prepared gradient echo also known as MPRAGE) sequence with *TR/TE* = 7.4/3.4 ms, matrix size = 208 × 227, α = 8°, 317 partitions, voxels size = 1.1 mm × 1.1 mm × 0.6 mm, *TI* = 868.7 ms. To prevent discomfort during MRI sessions, pads and air cushions were used to fix subject heads.

MR-compatible EEG caps with 64 electrodes (Compumedics Neuroscan Quik-Cap) were used for the entire experiment. In particular, the MR structural scan was acquired at the end of the neurofeedback experiment. Therefore, it should be noted that participants were inside the scanner (wearing the EEG cap) for around 1 h before extracting the MR volumes.

### Generating the Head Model

From T1W structural images each electrode can be seen directly as small bumps or protrusions over the scalp of the subject (see Figure 1). These protrusions are generated by the material of the EEG electrode and gel. Therefore, generating a head model which will include these protrusions is fundamental to localize the position of the electrodes. FreeSurfer (version 3.19; Dale et al., 1999)^1^ and Brainstorm software (version 5.30; Tadel et al., 2011) can be used to generate a head model directly from the anatomical MRI volume (Figure 2A). More importantly, when using Brainstorm this process is fast (less than 1 min to generate the head surface mesh using 10,000 vertices, erode factor = 0, and fill holes factor = 2). However, this step requires manual selection of fiducial points on the anatomical MRI volume to align the head model. This selection was done by an experienced user through the Brainstorm graphical interface.

### Electrode Positioning

The head model, available in Brainstorm, contains information about the curvature for each vertex in the 3D mesh. The position of the electrodes was found by isolating vertices with high curvature on the surface mesh of the head model. Using the coordinate system assigned by Brainstorm, we restricted a search space *z* > 0 in the axial plane. Since the coordinate system is based on fiducial points from the MRI image, by limiting our search to *z* > 0 we were able to exclude the vertices located on the nose, cheeks or lips of the head model. Next, we identified the 2,000 highest curvature vertices across the remaining mesh. This value considers the number of electrodes and characteristics of the EEG cap used in Sepulveda et al. (2016) experiment and may need to be changed to work in other systems (e.g., using more vertices to cover a higher number of electrodes). Due to the resolution of the mesh, there were groups of vertices with high curvature contained within the area corresponding to one single electrode. Therefore, from the group of 2,000 vertices selected above, we clustered the vertices whose distances from each other were within 1 cm (estimated diameter for the used electrode). Assuming that clusters containing the greatest number of vertices represent an electrode, we selected all the clusters that contained at least 10 vertices (Figure 2B). The centroid of each cluster was calculated to represent the position of the potential electrode (Figure 2C). A matrix containing the position of the potential electrodes was generated. Given that this step was not completely accurate (see section “Results”), a manual check of the potential electrodes were required. In most cases, an excess of points on the scalp was generated in the matrix; therefore, a manual removal of these extra points was performed by an experienced human analyzer on our team. Before moving onto the next step, a matrix of precisely 64 (unlabeled) electrodes was required. Please see the section “Localization” in the Supplementary Methods for more details on this stage. Custom MATLAB scripts were used for electrode positioning.

### Electrode Labeling Using the Distance Profile Method

**FIGURE 3:**
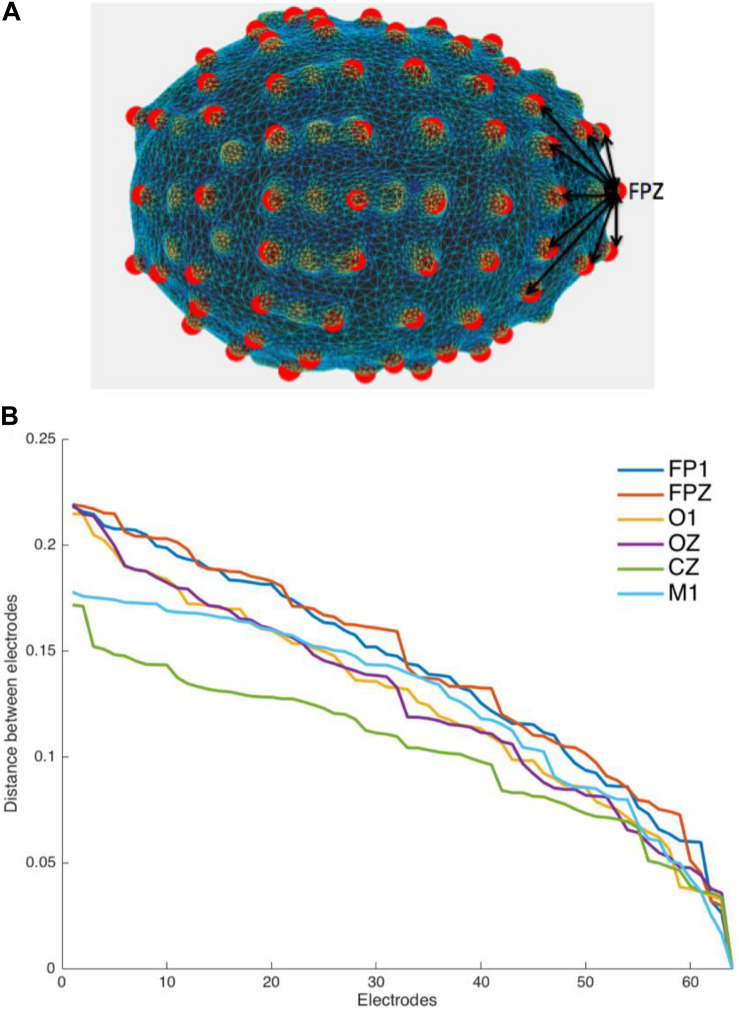
Distance Profile. **(A)** Distances from one electrode to other electrodes form a particular pattern that could be utilized in identifying and labeling electrodes. **(B)** The distance profiles for six of the 64 electrodes of a subject (frontal: FP1 and FPZ, occipital: O1 and OZ, and central: CZ and M1). To construct the distance profile, we set the Euclidean distances between the electrode of interest and the rest of the 63 electrodes in a descending order. Comparing the distance profiles of FPZ and OZ the total distance of FPZ from all other electrodes would decrease slower than in the case of OZ. This profile is used to individualize the electrode for the assignment of labels to each electrode by comparison with electrodes with known position and label (Note: Distance values were extracted from the units available through Brainstorm from the MRI volumes).

The next step was to correctly identify the labels of each of the 64 points that we located in the 3D head space. We hypothesized that each of the electrodes can be distinguished from one another based on their relative distances. For example, frontal electrodes like FPZ, FP1, or FP2 might have a greater number of electrodes far away from them than the number of electrodes close to them. In contrast, an electrode like CZ may have more electrodes closer to it than farther from it (Figure 3). This is mainly due to the general shape of the head and how the electrodes are arranged on the cap. For this reason, each electrode has a unique collection of Euclidean distances to all the other electrodes, otherwise referred to in this paper as their distance profile.

For each electrode, a distance profile was determined. To do this, we calculated the distances from an electrode to all the others, and we sorted these distances in a vector from the highest to the lowest (Figure 3). A crucial requirement for this step is having at least one template (i.e., a set of localized and labeled electrodes) with extracted reference distance profiles. Using this template, a Pearson correlation was calculated between the distance profiles of the unlabeled and template electrodes. The label of the template electrode was given to the unlabeled electrode with the highest correlated distance profile.

It should be noted that the distance profiles of electrodes located in symmetrical positions in the left and right hemispheres with respect to the central electrode axis (e.g., FP1 and FP2, C1 and C2, etc.,) might be identical. This created confounds for accurate labeling. To avoid this problem, we picked the electrodes labeled as FPZ and OZ to define a central plane. All electrodes that were located in symmetrical positions were compared against this central plane. A list with the pair of electrodes that are located in symmetrical positions with respect to the central plane was defined to run this comparison (e.g., FP1–FP2, F1–F2, and C1–C2, etc.). Once the unlabeled electrodes were each assigned a label using distance profile comparison, we checked if the hemisphere (right or left) was correctly assigned. For example, an electrode labeled as C1 could be C1 or C2. Since both C1 and C2 electrodes are in symmetrical positions, they should ideally have identical distance profile. If we found that this electrode was actually to the left side of the central plane, we kept its label as C1; however, if we found that it was actually to the right side of the central plane, we labeled it C2, the other symmetrical label. If pairs of electrodes are given the same label, the relative position of the electrodes are compared to determine which ones are located at the right and left hemisphere. In cases when more than three unlabeled electrodes receive the same name, a label was not assigned.

**FIGURE 4:**
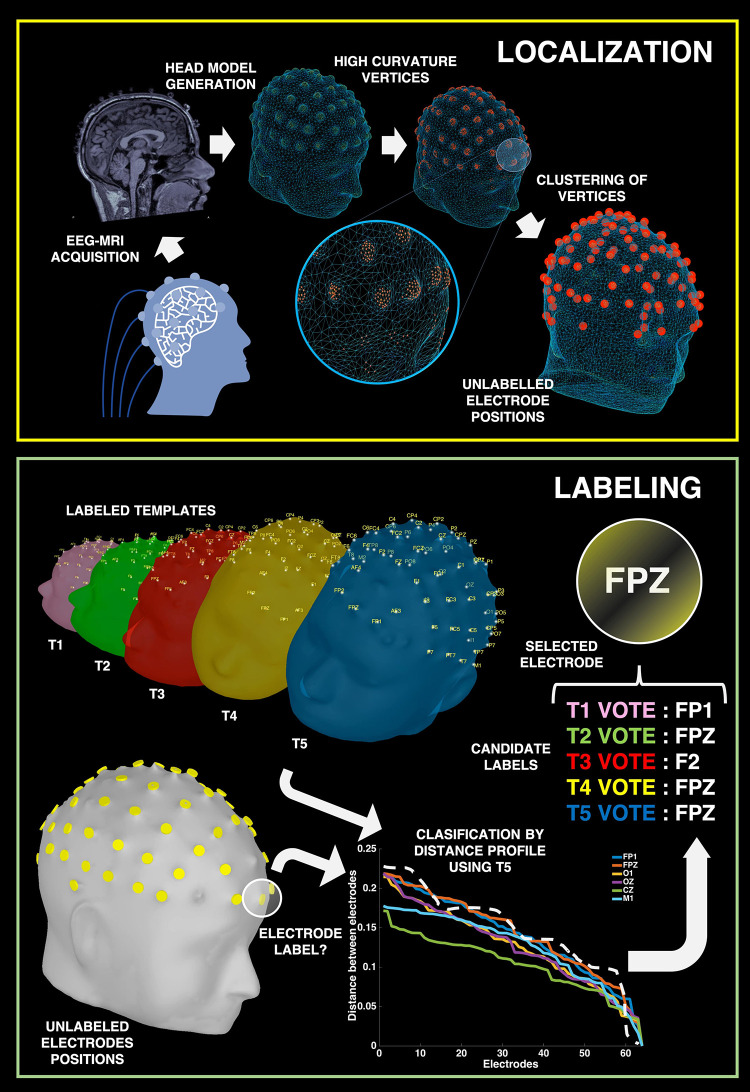
Processing Pipeline. Summary of processing pipeline for electrode localizations and labeling. Top: During EEG-fMRI acquisition, we extrapolate the location of each electrode by looking for protrusions across the subject’s scalp. Bottom: Through the use of labeled templates, we can classify the electrode by comparing the distance profiles for each unlabeled electrode (white dashed line) to the distance profiles of labeled electrodes of each template. The template analysis successfully votes to give the correct label of FPZ by 3 out of the 5 template votes.

In order to properly automate the process of labeling the electrodes, it is critical to use templates that are robust in defining the distance profiles for all the electrodes. However, given the wide variability between participants’ head shape, the use of a unique template may be inappropriate to arrive at correct electrode labeling. In our method, we used multiple templates for the labeling of individual electrodes to account for this variability. To estimate the improvement that the inclusion of additional templates provides to the labeling performance, we tested using 1, 3, and 5 templates. When multiple templates were used, we implemented a voting system for the definition of the final electrode label: Every template “proposes” a label for the unlabeled electrode and the label with most votes is assigned (see Figure 4). If there is no majority in the voting, the label that comes first in the sequential order of electrodes is assigned (e.g., the sequence for a 64-electrode Neuroscan Quik-Cap is presented in Supplementary Material). If none of the templates was able to identify a label for the electrode, the electrode remains unlabeled. For more details about this stage, please check “Labeling” section in the Supplementary Methods. Custom MATLAB scripts were used for electrode labeling.

### Detection Rate of Electrode Localization

To estimate the detection rate of our electrode localization method, we compared the results of our curvature positing algorithm with the true electrode positions. In our case, since we did not have access to digitizers or any other mechanism to extract the location of the electrodes, we determined the true position of electrodes by visual inspection of the locations in the MRI volume. In this way, points that did not correspond to electrodes [e.g., around eyes or ears, and electrooculogram (EOG) electrodes] were taken out. We used 26 structural T1W brain volumes for the purpose of testing the proposed method. We computed the detection rate of the method (i.e., true electrodes identified / 64) for each volume. A heat map was generated using EEGLAB, containing the information about the mean detection rate for the localization of electrodes across participants (Delorme and Makeig, 2004).

### Accuracy Analysis of Electrode Labeling

In order to check the accuracy of the distance profile method, we compare the labels assigned to the 64 electrodes by the algorithm with the true labels. To assess the performance of the method, we determined the value for true positives (TP, electrodes correctly labeled), false positives (FP, electrodes mislabeled), and false negatives (FN, electrodes for which no label was assigned).

We used the same 26 sets of 64 electrodes again as either templates or unlabeled electrode sets in order to test the accuracy of the labeling step. To test the accuracy and robustness of the multiple template approach, we considered different combinations of each of the 26 sets to generate variations of template groups. In other words, we ran multiple simulations in which we defined one of the electrodes sets as unlabeled, and picked 1, 3, or 5 of the remaining sets as templates. The vote system described above was used in each of the simulations for 3 and 5 templates. In the 5-template approach, due to the high number of possible combinations we selected only a subset to test (i.e., 30% of the total number of combinations was randomly selected). We calculated the TP, FP, and FN for every electrode set that was labeled using 1, 3, or 5 templates.

## Results

### Detection Rate of Electrode Localization Method

**FIGURE 5:**
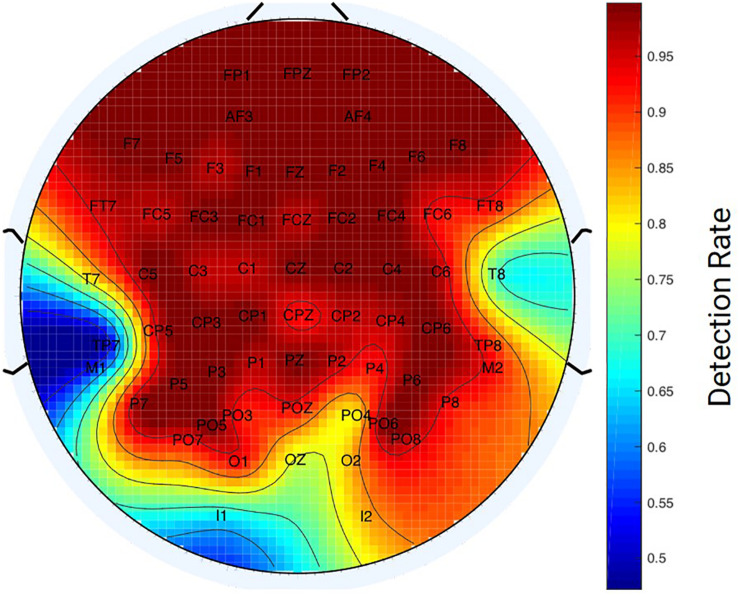
Detection Rate of Electrode Localization Method. Heat map of the mean accuracy for the localization of electrodes across 26 structural MRI scans. Areas indicated in red reflect high detection rate, while areas in blue reflect low detection rate for identifying electrodes.

From the structural MRI volume, we extracted the positioning of electrodes using curvature information from the vertices in the head model mesh. The centroid of the clusters of high curvature vertices were used to obtain the location of potential electrodes. Overall, the analysis of 26 different head models showed that our method was able to detect 93.99% of the electrodes [standard deviation (SD) = 0.0882] (Figure 5). From further inspection, we observed that the frontal and central electrodes were accurately identified by the algorithm (electrode detection over 90%). However, the localization of some occipital (e.g., OZ, I1, and I2) and lateral electrodes (e.g., T7 and T8) appeared to have a lower detection rate (electrode detection <80% accuracy).

On average, the algorithm identified 20.307 extra electrodes (SD = 3.8447), i.e., points indicated as electrode positions but without correspondence to real labeled electrodes. Considering that the caps had 64 labeled electrodes in total, this means the algorithm detects around 30% of excess points. This may be due to either artifacts in the MR-structural image, empty electrode holders in the EEG cap (dummy electrodes) or additional (e.g., REF, GND, and oculomotor electrodes). Importantly, the caps used in the experiment (Compumedics Quik-Cap) contained around 15 dummy or additional electrodes. Extra electrodes were removed manually to leave only 64 electrodes for the labeling step.

### Accuracy Analysis of Electrode Labeling

**FIGURE 6:**
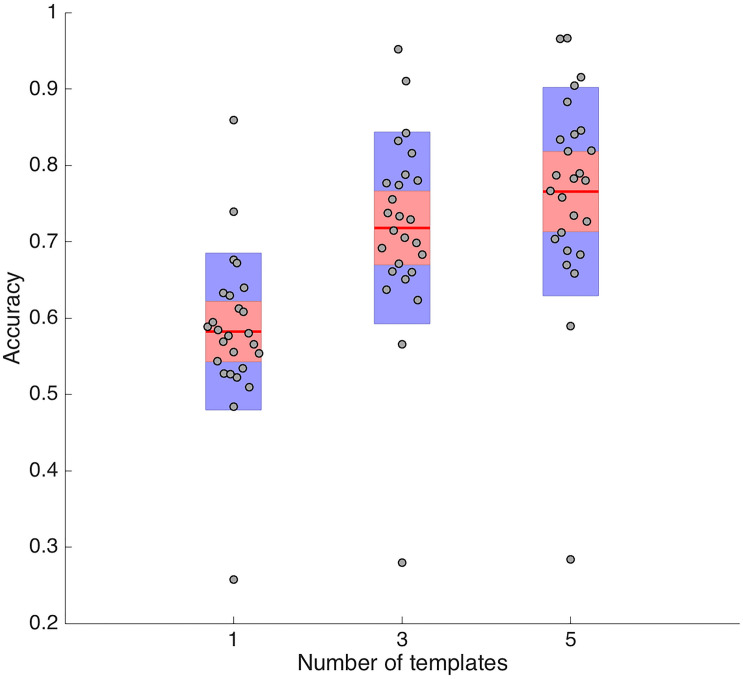
Electrode Labeling Accuracy. Box plots of the TP values for the different number of template(s): one template (left), 3 templates (middle), and 5 templates (right). The red line represents the mean value. Dots correspond to the accuracy obtained for each electrode set (26 in total). As the number of templates increases the mean accuracy also increases.

After locating the electrodes in the 3D head space, the Distance Profile method was employed to assign the labels. We used various templates (1, 3, and 5) to compare the electrodes’ distance profiles, as a way of generating more accurate and robust labeling. In the cases of 3 and 5 templates, a voting system determined the label to be assigned to each electrode. Due to a problem in determining the middle plane in the cap (missed labeling of FPZ and OZ), we were unable to run the algorithm in 2.314% of all the simulated unlabeled-labeled combinations set for one template approach (15 pairs out of 650 possible pairs). These pairs were excluded and TP, FP, and FN were calculated for the remaining combinations in the cases for 1, 3, and 5 templates. From the results of our simulations, we obtained the mean value for TP, FP, and FN for each one of the 26 electrode sets available (Figure 6 and Table 1).

**TABLE 1:** Summary of accuracy analysis with the distance profile method.

**Number of templates**		**True positives (%)**	**False positives (%)**	**False negatives (%)**
1	Mean value	58.23	20.05	21.72
	Standard deviation	10.27	9.05	3.96
3	Mean value	71.79	20.81	7.40
	Standard deviation	12.56	11.60	2.70
5	Mean value	76.55	20.83	2.62
	Standard deviation	13.65	13.21	2.12

The results in Table 1 show that as the number of templates increases there is a corresponding increase in the number of true positive electrodes and decrease in FN. Adding more templates increases the accuracy of labeling by enabling the cross-checking of labels and filling in of missing labels. Another trend that must be noted is that the number of FP remains at a constant value of about 20% despite increasing the number of templates.

**FIGURE 7:**
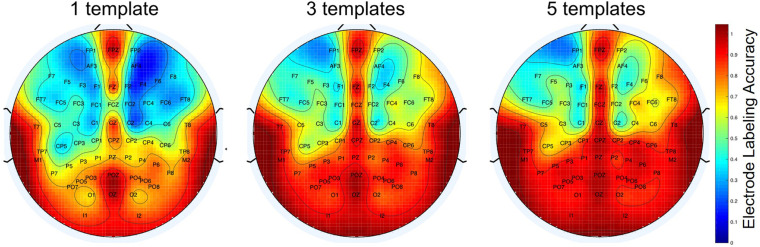
Electrode Labeling. Heat maps of the mean value of TP in the labeling of individual electrodes using the distance profile algorithm with 1, 3, and 5 templates. Red corresponds to a high accuracy of labeling while blue corresponds to a low accuracy. There is a positive relationship between the number of templates to the accuracy of labeling.

Additionally, we evaluated the accuracy of our distance profile algorithm by analyzing each electrode, pointing to identify the ones that are more difficult to label through this method. We generated spatial maps to display the accuracy of the method using 1, 3, and 5 templates (Figure 7). In this case, it appears that occipital electrodes are labeled with higher accuracy (>80% accuracy) while some fronto-lateral electrodes present more difficulties for identification (e.g., FP1 labeling accuracy <50%).

## Discussion

Localization and labeling of EEG electrodes are critical for the analysis of EEG data, especially for source reconstruction. Functional imaging in clinical applications (e.g., epileptic foci detection), neurofeedback and brain-computer interfaces (BCI) can greatly benefit from an accurate representation of the spatial location of the electrodes. Many methods have been proposed so far (Steddin and Botzel, 1995; Yoo et al., 1997; Le et al., 1998; Russel et al., 2005; Koessler et al., 2007; Péchaud et al., 2007; Marino et al., 2016; Butler et al., 2017; Clausner et al., 2017; Fleury et al., 2019; Homölle and Oostenveld, 2019; Taberna et al., 2019). However, most approaches require laborious manual intervention to prepare for the experiment or the use of special digitization devices.

The proposed approach enables the direct localization and labeling of EEG electrodes without the requirement of external devices (e.g., digitizers) in a context of simultaneous EEG-MRI experiments. Simultaneous EEG-fMRI experiments are already time-consuming and tedious for participants. The use of additional equipment (Steddin and Botzel, 1995; Brinkmann et al., 1998; Le et al., 1998; Lamm et al., 2001; Russel et al., 2005; Koessler et al., 2007; Clausner et al., 2017) and special protocols (Yoo et al., 1997; De Munck et al., 2011; Marino et al., 2016; Butler et al., 2017; Homölle and Oostenveld, 2019; Taberna et al., 2019) may increase setup time and cost, and cause fatigue and extra burden on the participants. Additionally, misplacement problems may arise between the electrode digitization (using external devices) outside the scanner and the later positioning of the participant inside the scanner. Also, our method does not rely on special MR acquisition sequences (Butler et al., 2017; Fleury et al., 2019), which may not be available in standard research or clinical setups. By using the spatial data provided from the individual MR structural volumes, it is possible to identify the electrodes on the head of a subject wearing a standard EEG cap. The MR structural volumes are required in any fMRI study for pre-processing. In this way, we were able to preserve the position in which the fMRI experiment was performed without incurring any extra time and cost than required for the standard fMRI procedure.

In the initial step of localization of the electrodes, we utilized the curvature data given by the head model to find the Cartesian coordinates of the electrodes. Protrusions with high curvature are formed in the surface of 3D reconstruction of structural scans, due to the volume of gel contained between the electrode and the scalp. Using this method over 90% of the electrodes were accurately localized in our simulations. Our results found that the occipital and temporal electrodes were more difficult to localize than electrodes in other regions. This outcome could be due to a decrease in the MR signal resulting from the gels in those electrodes. It should be noted that the structural MR volumes were acquired at the end of the experiment; thus, the subjects had been inside the scanner in a supine position for around 1 h. Since the EEG electrodes were prepared by gelling at the earliest time in the preparation for the experiment, it is possible that the gel would have dried considerably by the time of the MR acquisition. The regions with poorer localization are the ones in the posterior electrodes, which coincidentally are the electrodes over the which the participant’s head rests during the experiment. One cause for this might be that these regions are drying faster because they may experience higher temperatures during the experiment. Another potential cause is that the gravitational force can displace the gel away from the scalp in these posterior electrodes when the subject is in the supine position. Additionally, hair presence may increase the space between the electrodes and the scalp, causing the gel to spread out from the electrode capsule. By detaching part of the volume off of the scalp surface, these complications decrease the signal observed in those particular electrodes. These changes in the electrode gel throughout the course of the scan can lead to variation in the localization across subjects. Such errors may lead to mislabeling problems, as a result of variability in the distance profiles of the electrodes. To overcome the above problems, we propose that the MR structural images are acquired at the beginning of the experiment when the gel is fresh and is contained in all electrodes to an equal extent.

During automated labeling, our method distinguishes each electrode based on its particular distance profile: The descending ordering of the distances between each electrode and the other 63 electrodes in the cap. Given that each electrode has a distinct location, the distance profile of each will be different from other electrodes in the same hemisphere to allow for proper labeling. To carry out the labeling, we used the distance profiles of previously labeled electrodes as templates for comparison. As observed above, the use of multiple templates results in higher accuracy of the labeling: From an average of 58% of electrodes correctly labeled for 1 template to a 76% for 5 templates. Through the use of multiple templates, many of the false negative electrodes were reduced. Interestingly, although the number of FN reduces with the use of multiple templates, the number of FP (i.e., electrodes that were mislabeled) did not reduce significantly. Since this number seems to be independent of the number of templates we are using, it may be related to a particular weakness of our distance profile comparison. A higher similarity of the distance profile in a particular subgroup of electrodes might make it difficult to distinguish electrode identity using correlations; thus, hindering successful classification. Therefore, the same label would be assigned indistinctly to different electrodes, generating mismatches. We think this is the case for the lateral frontal electrodes.

Despite the increase in the accuracy of labeling with templates, it is observed that lateral frontal electrodes are the most difficult to identify using this method. As mentioned above, similar distance profiles between the electrodes in this region may generate FP (e.g., FC1 has a very similar profile to C1, making them prone to labeling mismatch). Additionally, we think this problem may arise from the way our method deals with symmetry of electrode locations on left and right hemispheres (e.g., F1 and F2 electrodes). In our method, to identify electrodes that are located at symmetrical positions on the cap we define the midplane using frontal and occipital electrodes located in the center. In the case that two electrodes are assigned the same label we assumed that they may correspond to a symmetrical pair (e.g., two electrodes labeled as C1 when they actually should be C1 and C2). To distinguish them, we considered the location of each one of them relative to the midplane. However, if we have three electrodes assigned with the same label, we decided to keep them unlabeled. The reason for this was to avoid having electrodes with the same name in the cap since it would cause confusion as to which of the three electrodes belong to the symmetrical pair. Therefore, it is possible that if the frontal electrodes have similar distance profiles, they might be given the same label (more than twice). This would cause those electrodes to remain unlabeled.

We can compare our values on detection rate and labeling accuracy to the most recent methods that use EEG-fMRI system-based approaches. When looking at Fleury et al. (2019) and Butler et al. (2017), who present an approach that does not require any additional devices, we see that they report a detection rate around 94%. Our detection rate is similar to their results. One disadvantage of their approach is that they did not contribute to solving the problem of labeling and relied on using De Munck’s Pancake model (2011). For this reason, these papers did not present any values on localization accuracy; thus, we could not compare our results with those. We also compared our results with Marino et al. (2016) and De Munck et al. (2011), whose papers present a semi-automated method to solve the two-step problem. When comparing our results to De Munck et al. (2011), we saw that their approach had a manual selection process for localization of electrodes. This is decidedly a big limitation since it requires relatively more time and an experienced user to manually select each electrode. In our study, the localization step is automated, although in practical terms minor human intervention is required to correct detection errors. Marino et al. (2016) presents an automated approach that has a very good detection rate and labeling accuracy. While their approach had very few FN detected in the localization step (<0.5%), they also had FP detected (∼16% of 256 electrode set, which is even further reduced using additional filtering). Our false positive rate was higher than theirs (∼30% of 64 electrode set). However, please note that our false positive rate for detection does not take into account the fact that some of these FP are caused by DUMMY or REF electrodes (i.e., extra electrodes physically present in the cap). Their labeling step notably yielded no FP or FN. We found that our labeling accuracy was ∼77% when using 5 templates. While Marino et al. (2016) present a more accurate approach for solving this two-step problem, it is based on the use of high-density electrodes (HydroCel Geodesic Sensor Net, Electrical Geodesics) for proper localization and labeling, which is not a standard EEG system in EEG-fMRI studies.

The labeling accuracy of our method relies on the following assumptions: (1) head shapes and distance profiles are similar across the subject population and (2) the localization of the electrodes is performed using the standard 10–20 system. However, it is known that head morphology is variable across humans, e.g., relationship between head circumference and height (Bushby et al., 1992). Additionally, the use of the same EEG caps in heads of variable shapes unavoidably will lead to discrepancies in electrode positions. We must also consider that our method uses individual head models as templates, making the identification even more idiosyncratic. To ameliorate these variabilities, one might consider using average head models or probabilistic head models. Furthermore, the method might be improved by generating a more sophisticated voting system when multiple templates are used. Statistical measures such as t-maps can be used. Also, to avoid assigning the same label to different electrodes, it might be useful to utilize the information of nearby electrodes to help characterize an electrode’s identity more precisely (e.g., include in the comparison of distance profiles not only the electrode to label but also neighboring electrodes).

In our method, the localization and labeling steps are designed as automated procedures. However, between localization and labeling user supervision is required to remove (or add) localized positions from the electrode matrix when more (or less) than 64 points are reported. As mentioned earlier, the identification of high curvature areas around the ears or additional electrodes (e.g., REF electrodes) are some of the reasons for these FP. In our experiments, empty electrode holders were located mostly in the occipital region to distribute head weight. The inclusion of these electrodes as part of the labeling step (e.g., as DUMMY or REF) may be a solution to reduce the human intervention required at this stage.

In conclusion, here we have presented a semi-automated and direct method for localizing and labeling electrodes on a standard EEG cap, from MR structural images, acquired during simultaneous fMRI-EEG experiments. We used elements required for standard EEG-fMRI studies (i.e., T1W structural scans and standard MRI-compatible EEG caps using conductive electrode gel), presenting a more economical approach to the two-step problem of labeling and localizing electrodes. The method exploits data that is already available (MR structural scans); thus, avoiding the extra time and cost that is otherwise involved in the use of external digitization devices and cumbersome manual processes or extra modifications to the standard EEG-fMRI procedure. Our future work will conduct comparisons of this method with other existing approaches.

## Data Availability Statement

The code used for the localization and labeling of electrodes is available in GitHub: https://github.com/pradysepulveda/EEG_Loc_Label_2020. Example electrodes sets are also available.

## Ethics Statement

The experimental protocol was approved by the ethics committee of Pontificia Universidad Católica de Chile. Each participant signed a written informed consent during the study.

## Author Contributions

AB, PS, SR, and RS designed the study and methodology. PS acquired MRI scans of participants. PS and AB analyzed the data. AB, PS, SR, RT, TO, and RS worked on the discussion and wrote the manuscript. All authors contributed to the article and approved the submitted version.

## Conflict of Interest

The authors declare that the research was conducted in the absence of any commercial or financial relationships that could be construed as a potential conflict of interest.

## Footnotes

1 http://surfer.nmr.mgh.harvard.edu/
