# Supplementary material for: Semi-Automated and Direct Localization and Labeling of EEG Electrodes Using MR Structural Images for Simultaneous fMRI-EEG
Source: Front Neurosci. 2020 Dec 22;14:558981. doi: 10.3389/fnins.2020.558981 (PMC7783406; doi:10.3389/fnins.2020.558981)
Supplement: Supplementary file 1 [file Data_Sheet_1.PDF]

Semi-automated and direct localization and labeling of EEG electrodes using MR structural images for simultaneous fMRI-EEG.

(SUPPLEMENTAL METHODS)

Abhishek Bhutada[1, *], Pradyumna Sepúlveda[2, *], Rafael Torres[3], Tomás Ossandón[3,5], Sergio Ruiz[3,4,#] and Ranganatha Sitaram[3,4,5#]

1. University of California, Berkeley, USA
2. Institute of Cognitive Neuroscience, University College London, London, England
3. Department of Psychiatry, Faculty of Medicine, Interdisciplinary Center for Neuroscience, Pontificia Universidad Católica de Chile, Santiago, Chile
4. Laboratory for Brain–Machine Interfaces and Neuromodulation, Pontificia Universidad Católica de Chile, Santiago, Chile
5. Institute for Biological and Medical Engineering, Pontificia Universidad Católica de Chile, Santiago, Chile

* Equal contribution
# Corresponding authors' emails: rasitaram@uc.cl , sruiz@uc.cl

A. Supplemental Methods

1. Localization

a. *Generate head model*: Brainstorm (version 3.19; Tadel et al. 2011) was used in this example. Initially an anatomical MRI volume (non-normalized) was loaded and fiducial points were identified (Figure S1A). This allows the identification of the head space. Brainstorm allows the direct generation of a three-dimensional head surface from the MRI volume (Figure S1B). On the generated head mask (Figure S1C), it is possible to visualize the electrodes as protuberances on the surface. From Brainstorm menu, surface information was exported to Matlab workspace (Figure S1D) as a structure containing, among other relevant variables, the position of vertices generating the surface and its curvature (Figure S1E). It was possible to visualize this as a three-dimensional mesh (Figure S1F).

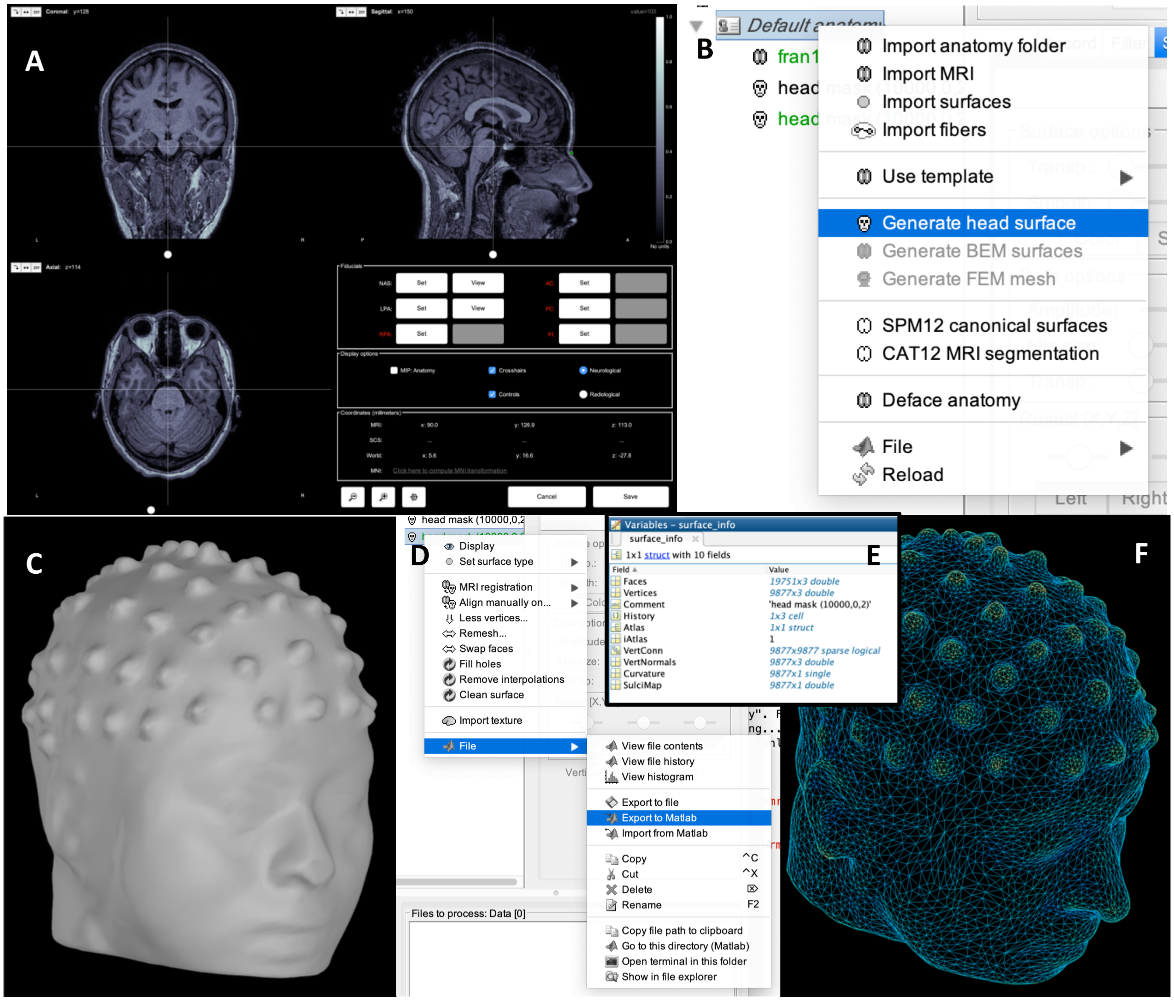

Figure S1. *Stages for the generation of the head model.*

b. *Curvature selection*: All the vertices were ordered from highest to lowest curvature. To constrain the number of possible vertices, all the vertices with z < 0 (z corresponded to the craniocaudal axis) were discarded (Figure S2A). This was done to avoid picking high curvature vertices around nose and lips. We set a treshold for the number of vertices (*num_tops* = *2000*) with highest curvature to be picked (B). This variable was selected considering our configuration and it may need to be adjusted for the type of cap and number of electrodes (Figure S2B). In Figure S2C, we show how modifying the parameters for *num_tops* affects the number of electrodes that are localized. It is important to notice that while the number of electrodes identified (*hits*) increases when the number of vertices increases, the number of non-electrodes vertices also increases.

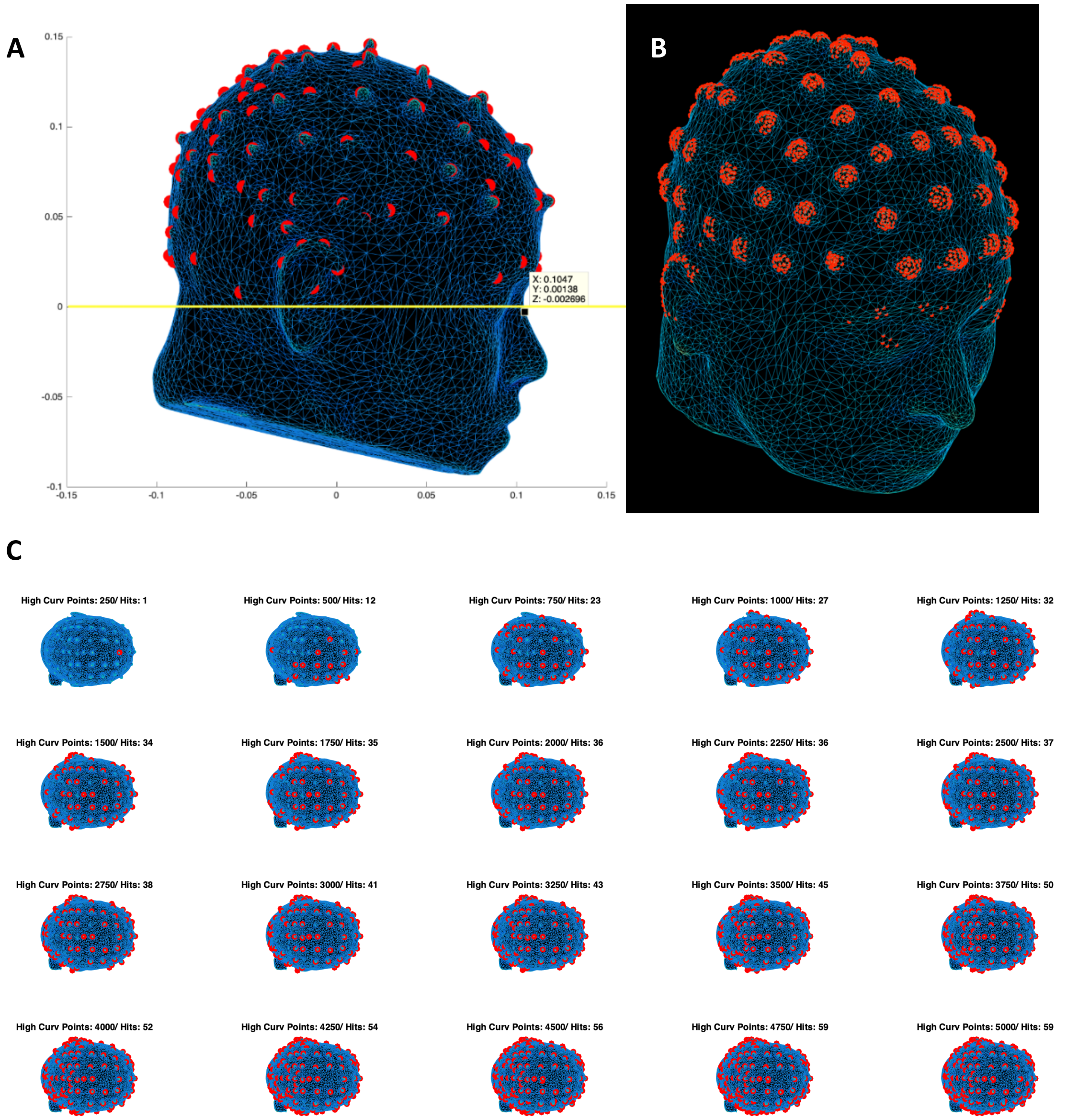

Figure S2. *Stages for curvature selection and potential electrodes positions. (A) The search space of high curvature points was constrained on the* craniocaudal *axis (z>0). (B) Highlighted vertices (in red) correspond to high curvature points (num_tops = 2000 vertices). (C) Variation of the threshold on high curvature points (i.e. num_tops) affects the number of electrodes that are selected for one subject.*

c. *Vertices clusters*: Considering the subset of vertices selected in the previous step, a custom clustering process was implemented to define the position of potential electrodes. One electrode might contain multiple high curvature points. All the high curvature vertices that were within the diameter of one electrode (aproximate 1cm, which was defined manually after measuring the diameter of an electrode in the head space) were set to belong to the same "cluster". Therefore, the algorithm checked each vertex in the set and estimated the Euclidean distance to the other vertices: if the distance was shorter than the diameter of an electrode, then both vertices were assigned to the same cluster. If a vertex remains unassigned after estimating the distance to all the other vertices, then a new cluster is created. Once all the vertices were grouped (Figure S3A), the clusters with more than 10 vertices in it were assumed to be potential electrodes. The centroid of the vertices in a cluster was set as the three-dimensional location of that potential electrode (Figure S3B). Since it was usual that the number of points generated was higher than the total number of electrodes (64), in our case human inspection was required for the removal of the excess of positions.

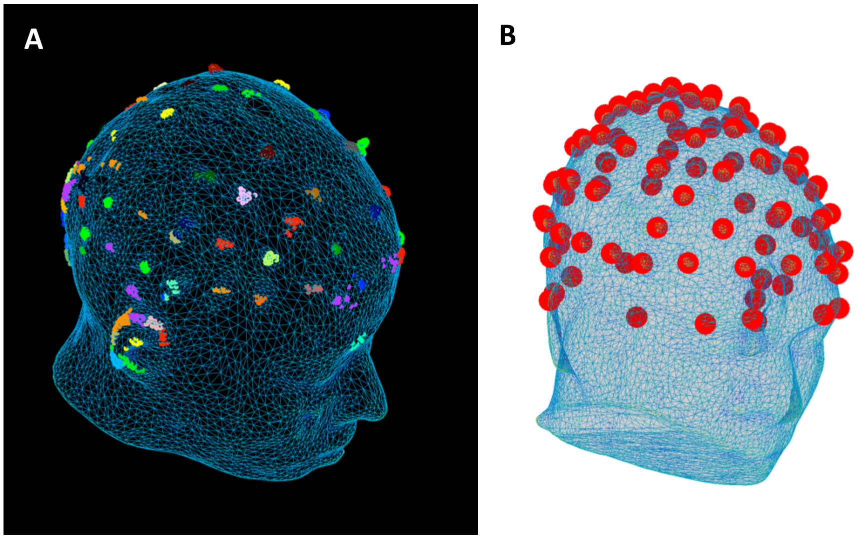

Figure S3. *Clustering of vertices location to generate potential electrode positions. (A) Different colors indicate clusters of vertices generated using our algorithm. (B) Centroid of the clusters with more than 10 vertices in them are shown as the positions for the potential electrodes.*

2. Labeling

a. *Generate distance profiles*: At this stage, a matrix with the unlabeled electrodes positions (three-dimensional coordinates obtained from the previous step) and a template matrix with the position of electrodes properly labeled should be available. Both matrices must have the total number of electrodes (64 for our tests). For both matrices, we calculated the Euclidean distance between each electrode and all the others. For each electrode the distances were sorted from the highest to the lowest which we referred as the *distance profile* for that electrode (Figure S4). Two separate matrices (each one of size 64x64) contained the distance profiles for the unlabeled and template electrodes.

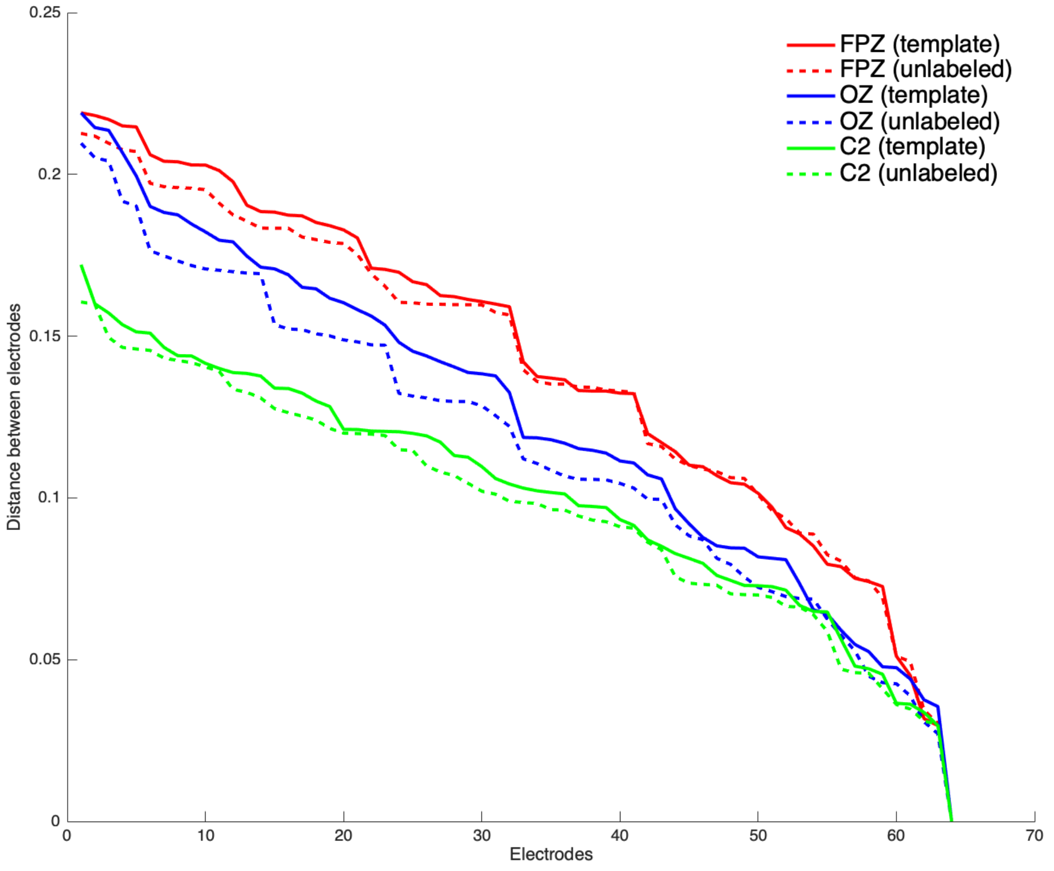

Figure S4. *Distance profile estimated for 3 different electrodes (FPZ, OZ and C2) in two sets.*

b. *Assign labels based on distance profiles*: Pearson correlations were calculated to compare the distance profiles of the unlabeled electrodes with the profiles of all the electrodes in the template matrix. The label of the electrode in the template with the highest correlation was assigned to the unlabeled electrode. In the first check, we only kept those electrodes that were assigned only one label. Since the distance profile for electrodes in symmetric locations of the cap might be identical (e.g. C1 and C2), determining the final label requires confirmation of the side in which the unlabeled electrode was located (i.e. left of right hemisphere assuming the head is facing forward on the x-axis). For this purpose, we used the position of FPZ and CZ, labeled using distance profile correlations, to generate a plane that divides the Y-axis in the two hemispheres, right and left (Figure S5). To generete a plane from two points in the three-dimensional space, the midplane was constrained to be perpendicular to the X-Z plane. If the label of an electrode did not correspond to their location according to the plane (e.g. C2 appeared located to the left of the midplane), then that electrode was set as the symmetric electrode (e.g. mislabeled C2 electrode was assigned to C1). In a second check, we considered the cases in which the same label was assigned to two electrodes. Here, we assumed they belonged to a symmetric pair (e.g. if two electrodes receive label CP5 that means one of them is CP4 and the other CP5), then they were relabeled according to their relative position (e.g. of the two electrodes the one located in the rightmost position on the horizontal axis was labeled as CP4). When the same label was assigned 3 times or more, those electrodes remained unlabeled. A new matrix (*correct_labels*) was generated in which each row corresponded to one of the 64 electrodes and the 3 columns contained the x,y,z coordinates for the position. Labels that were not assigned to any electrodes were reported as [0,0,0].

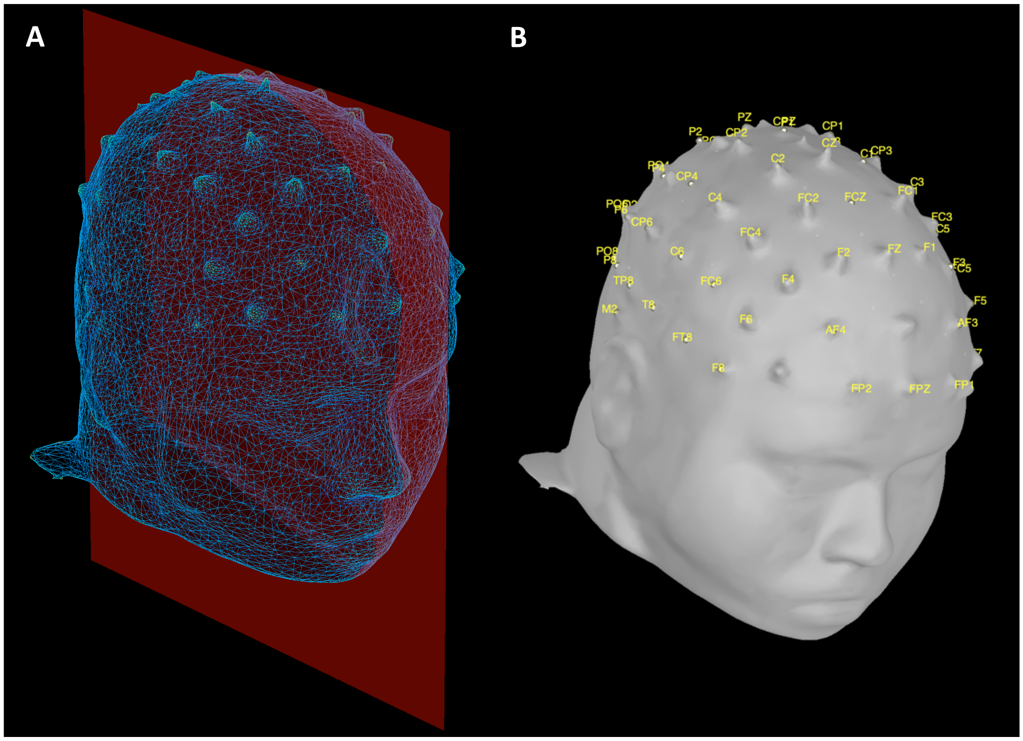

Figure S5. *Electrode labeling process. (A) A midplane constructed from the position of FPZ and CZ electrodes was used to separate left from right side electrodes labels. (B) Final labeling of the electrodes.*

Note: The labelling process described above was repeated for 1, 3 and 5 templates. When multiple templates were used, the labeling process described above is repeated for each individual template generating multiple *correct_labels* matrices. The voting process was performed for each electrode individually considering the labels indicated in each one of the *correct_labels* matrices.

If additional details of the method are required please contact the authors.

B. Example EEG electrodes position file (.xyz files)

Electrode Number	X	Y	Z	Label
1	-0.02371932	0.108779592	0.062907857	FP1
2	0.002414829	0.114552769	0.060506097	FPZ
3	0.02812587	0.107277797	0.060958972	FP2
4	-0.02961334	0.09544877	0.079220874	AF3
5	0.032698451	0.090666045	0.084430314	AF4
6	-0.07155935	0.066298681	0.056566761	F7
7	-0.06129487	0.065955685	0.080138486	F5
8	-0.04149525	0.071426761	0.098626268	F3
9	-0.02271496	0.073518511	0.109109916	F1
10	0.003938555	0.07578517	0.112739755	FZ
11	0.025335496	0.073234195	0.108494816	F2
12	0.045390672	0.069274628	0.100123275	F4
13	0.065879364	0.061249377	0.081778486	F6
14	0.074606479	0.056688727	0.054580848	F8
15	-0.08082581	0.040714092	0.056882659	FT7
16	-0.07152277	0.04488985	0.083453267	FC5
17	-0.05320574	0.048567121	0.106647676	FC3
18	-0.0292698	0.052461397	0.121635259	FC1
19	0.003059592	0.053728763	0.12819895	FCZ
20	0.032950392	0.048699591	0.122976761	FC2
21	0.05852035	0.042746974	0.107932963	FC4
22	0.077545163	0.037099739	0.083197844	FC6
23	0.082633006	0.030396682	0.050424357	FT8
24	-0.0844807	-0.02057882	0.039696038	M1
25	-0.0838768	0.012894536	0.048299088	T7
26	-0.07790395	0.018970446	0.083910883	C5
27	-0.06069117	0.022596447	0.110869964	C3
28	-0.0321293	0.023228153	0.131056472	C1
29	0.001297203	0.023354983	0.141849604	CZ
30	0.031710124	0.018602108	0.133196919	C2
31	0.059180372	0.015496683	0.116039578	C4
32	0.080776148	0.010229073	0.087614109	C6
33	0.081185075	0.005585733	0.052592023	T8
34	0.083240552	-0.02663351	0.043629179	M2
35	-0.08137458	-0.01194787	0.055151195	TP7

36	-0.06168996	-0.0094946	0.114162044	CP3
36	-0.07895113	-0.01328471	0.083807222	CP5
38	-0.03174812	-0.00840547	0.133690709	CP1
39	-0.00013928	-0.01043891	0.140111131	CPZ
40	0.03077305	-0.01562496	0.134727103	CP2
41	0.058757322	-0.02025817	0.116774055	CP4
42	0.075276783	-0.02762503	0.089129675	CP6
43	0.081287093	-0.02892743	0.060201125	TP8
44	-0.07841584	-0.03829836	0.058516973	P7
45	-0.07259932	-0.03967876	0.083934621	P5
46	-0.05491297	-0.04084599	0.109292432	P3
47	-0.02776125	-0.04315873	0.123989822	P1
48	-0.00071671	-0.04556218	0.127458344	PZ
49	0.025811582	-0.04836311	0.123276477	P2
50	0.048551069	-0.05056011	0.109179127	P4
51	0.066128691	-0.05022303	0.086326545	P6
52	0.071650036	-0.05228846	0.056723749	P8
53	-0.06542206	-0.06193709	0.060206172	PO7
54	-0.05646068	-0.06553826	0.079318217	PO5
55	-0.04259684	-0.06843593	0.092027144	PO3
56	-0.00382126	-0.07326609	0.103688289	POZ
57	0.032423635	-0.07659565	0.090456654	PO4
58	0.049349165	-0.07372291	0.07550995	PO6
59	0.056780769	-0.07150408	0.054213634	PO8
60	-0.0383139	-0.08351854	0.064060206	O1
61	-0.00177229	-0.09159482	0.068581322	OZ
62	0.029571092	-0.088949	0.061194599	O2
63	-0.03803986	-0.08438164	0.027945436	I1
64	0.026052944	-0.08490675	0.028723318	I2

References

Tadel, F., Baillet, S., Mosher, J. C., Pantazis, D., and Leahy, R. (2011). Brainstorm: a user-friendly application for MEG/EEG analysis. *Comput. Intellig. Neurosci.*
